# 

**Published:** 1858-08

**Authors:** 


*The Ohio Medical and Surgical Journal.*—We notice that the July No. of this valuable journal appears with another name on the cover, Dr. Dawson having associated with him Prof. J. W. Hamilton, Prof. of Surgery in the Starling Medical College.

---

*Medical Society of South-Western New York.*—This society will hold its next meeting at the Jamestown House, Jamestown, on the 4th inst. A good address may be expected from Dr. Hazeltine, and a good time generally.

---

*Not too Late.*—We have yet a few spare copies of Vol. XIII, which we offer to any one gratis, who will send us his name and $2.50. We are not quite exhausted, but soon it will be too late to take advantage of this offer.

---

*What may Happen.*—We are afraid that we shall have to dun in our next number. It will be only dire necessity which will drive us to the alternative of asking for our due, therefore we beg those indebted to us to communicate with Mr. E. R. Jewett, Buffalo, and spare us the humiliation.

